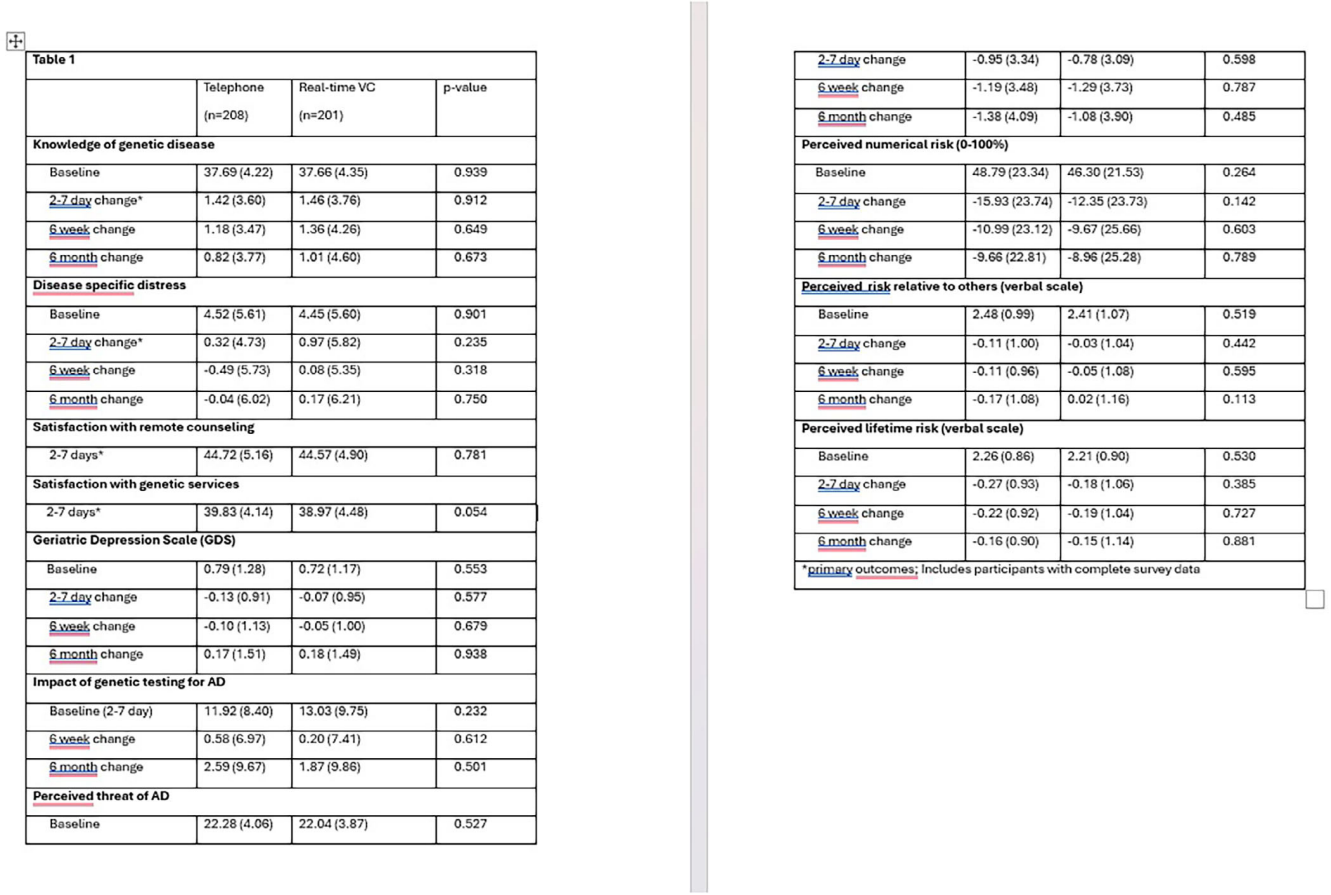# CONNECT 4 *APOE*: A randomized study of phone versus videoconference delivery of *APOE* genotype disclosure in the Generation Study

**DOI:** 10.1002/alz70858_100327

**Published:** 2025-12-24

**Authors:** Angela R. Bradbury, Brian L Egleston, Elisabeth Wood, Jason Karlawish, J. Scott Roberts, Scott Kim, Cara Cacioppo, Laura Eppelmann, Michelle Weinberg, Demetrios Ofidis, Carolyn Langlois, Eric M. Reiman, Pierre N. Tariot, Jessica B. Langbaum

**Affiliations:** ^1^ University of Pennsylvania, Philadelphia, PA, USA; ^2^ Fox Chase Cancer Center, Philadelphia, PA, USA; ^3^ University of Michigan School of Public Health, Ann Arbor, MI, USA; ^4^ National Institutes of Health, Bethesda, MD, USA; ^5^ Banner Alzheimer's Institute, Phoenix, AZ, USA

## Abstract

**Background:**

*APOE* genotyping is increasingly important to identify candidates eligible for Alzheimer's prevention trials. Such testing is also now commonplace for patients considering new anti‐amyloid treatments, given that *APOE4* carriers are at elevated risk for treatment side effects. Data to inform best practices for sharing *APOE* results are needed.

**Method:**

CONNECT4 is a multi‐center, randomized trial (NCT02978729) comparing disclosure of *APOE4* genotype by remote videoconference (VD) or telephone (TD) with a genetic counselor in cognitively unimpaired adults 60‐75 YO. It was ancillary to the Generation 1 prevention trial, enrolling at 20 US sites. Patient‐reported outcomes (PROs) were collected at 2‐7 days (T1), 6 weeks (T2), and 6 months (T3) following *APOE4* disclosure. Primary outcomes were change in genetic knowledge and disease‐specific distress (T0‐T1) and satisfaction (T1). We used T‐tests and Fisher's exact tests to compare arms and carrier groups.

**Result:**

274 participants were randomized to TD and 267 to VD. Of those with complete data (*n* = 409, 76%), the mean age was 67.1 YO, 64.1% were female, 92.2% were white; 77.8% reported a family history of AD and 29.8% were *APOE4* homozygotes, 40.4% were heterozygotes and 29.8% were noncarriers. There were no significant differences among arms in knowledge or distress, but slightly higher satisfaction with TD (Table 1). There were no significant differences in secondary PROs. By genotype, the only differences by arm included change in perceived lifetime numerical risk from T0‐T1 among homozygotes (TD ‐5.19 v. VD +4.07, *p* = 0.018), and T0‐T2 among noncarriers (TD ‐13.33 v. VD ‐22.28, *p* = 0.053) and satisfaction with genetic services among heterozygotes (TD 40.46 v VD 38.65, *p* = 0.01). In analyses evaluating moderators of PROs that adjusted for result, state anxiety increased under TD and decreased under VD for non‐White participants (T0‐T1), but declined by a similar amounts under both modalities for White participants (*p* = 0.039).

**Conclusion:**

Both telephone and videoconference are reasonable options for disclosure of *APOE* results in cognitively unimpaired individuals across all genotypes. While satisfaction was slightly higher with TD, small differences in numerical perceived risk by genotype and reductions in state anxiety among non‐white participants, suggest potential small benefits to VD for some subgroups.